# The Effect of Commuting Time on Quality of Life: Evidence from China

**DOI:** 10.3390/ijerph20010573

**Published:** 2022-12-29

**Authors:** Libin Han, Chong Peng, Zhenyu Xu

**Affiliations:** 1 Economic and Social Development Research Institute, Dongbei University of Finance and Economics, Dalian 116025, China; 2 School of Economics, Nanjing Audit University, Nanjing 211815, China; pengchong@nau.edu.cn; 3 School of Statistics and Data Science, Nanjing Audit University, Nanjing 211815, China

**Keywords:** commuting time, quality of life, transport, smart city

## Abstract

This study examines the effect of commuting time on quality of life. We find that the longer the commute time workers use, the lower satisfaction with work and life they have; the long commute also causes health damage, affecting physical health and causing inactivity. However, better public transportation infrastructure can decrease commuting time, especially the construction of subways.

## 1. Introduction

In 2020, more than 56.16% of the world’s population lived and worked in cities (https://ourworldindata.org/urbanization#number-of-people-living-in-urban-areas, accessed on 26 December 2022). Due to this concentration of the population, cities are facing increasingly serious traffic and congestion problems. People living in cities often need to travel for work because of the job–housing imbalance. Commuting time occupies a large proportion of their daily time. On average, workers spend 10.5% of the time available for work and travel on commuting, which corresponds to a 28 min single trip for an 8 h workday [1]. 

Previous studies showed that there is a commuting time paradox: the commuting time, on average, does not change largely over time and space [1,2]. The ‘co-location hypothesis’ states that residents and workers will change their residence or workplace, or both, to adapt to worsening congestion, and when they change their location, this results in the average commute time being stable [3]. 

However, recent survey data show that the average commuting time is increasing. Commute time in the United States has been on the rise nationwide since 2010. Survey data show that the average one-way commute in 2016 crept up to 28 min, from 26.6 min in 2015 (*How the American commute has changed over the past 50 years*, https://getjerry.com/newsroom, accessed on 7 February 2022). Moreover, the difference across individuals is large [4].

According to the Alonso–Muth–Mills spatial model [5,6,7], the increase in commuting time is offset by a higher wage and greater demand for housing. The burden of commuting is compensated so that individuals’ utility is equalized. However, commuting has been shown to have negative consequences for workers. People with longer commuting time report systematically lower subjective well-being [8]. Clark et al. [9] found that an additional 20 min of commuting each working day is equivalent to a 19 percent annual pay cut. Shorter commute times and walkable commutes can improve well-being.

City residents need public transportation to support their daily activities. There has been an aggravation of urban air quality deterioration due to urbanization, transportation, and economic development, which leads to damage to health [10]. Researchers have recognized the connection between public health and transportation [9]. However, existing research mainly focuses on developed countries, and there have been few studies on developing countries. In fact, developing countries are undergoing continuous urbanization; however, their construction of transportation infrastructure lags in relative terms, so the commuting problems residents face need be studied further. Accordingly, the object of research in this study is the biggest developing country, China, with a focus on the impact of commuting on the quality of life in China’s urbanization process. This impact includes not only life satisfaction but also health and other factors. From the policy perspective, the role of public transportation and big data in mitigating commuting time and improving the quality of life is also discussed.

Our research found that the extension of commuting time has a negative impact. The longer the commute, the lower the satisfaction with work and life; the length of commuting can also cause damage to health, affecting physical health and causing inactivity. However, the increase in public transportation, especially the construction of subways, can ease commuting time. 

The rest of the paper is organized as follows: Section 2 is a review of related research on commuting and quality of life. Section 3 empirically analyses the relationship between commuting and quality of life. The last section concludes.

## 2. Literature Review

Our paper is related to study on commuting within a city. Existing research suggests that excessive commuting time can have a negative effect. Huang et al. [11] found that commuters whose travel time exceeds 45 min prefer to shorten commutes via moves, while others with shorter commutes tend to increase travel time in search of better jobs and/or residences. Commuting between home and work is routinely performed by workers, and any impact of commuting on well-being will consequently affect a large proportion of the population. Data from the US showed that more time spent on the daily commute was related to higher levels of fatigue and stress during commuting [12]. Clark et al. [9] found that longer commute times were associated with lower job and leisure time satisfaction, increased strain, and poorer mental health. Christian [13] showed that longer commutes were associated with behavioral patterns that over time may contribute to obesity and other poor health outcomes. Subjective health measures are clearly lower for people with longer commutes in the UK [14]. Using data from the Panel Study of Income Dynamics for the years 2011 to 2017, we found that a 1% increase in the daily commute of workers was associated with an increase of 0.018% and 0.027% in the days of sickness absence per year for male and female workers, respectively [15]. The benefits of reducing commuting costs are obvious. Monte et al. [16] found that reductions in commuting costs would generate welfare gains of around 3.3 percent.

Commuting has significant differences in different industries and households. Secondary-sector workers tend to reside near their workplaces because of relatively balanced jobs and housing, whereas tertiary-sector workers tend to reside further away from their workplaces to save housing costs [17]. Low-income workers have the shortest commutes due to the location of informal work activities. Men commute longer than women [18,19]. Labor force participation rates of married women are negatively correlated with the metropolitan area commuting time [20]. 

This paper is also related to the literature on transportation infrastructure. The construction of transportation infrastructure, especially the construction of public transportation facilities, can significantly improve mobility in cities and reduce the commuting time of cities. Increasing transport mobility and favorably altering perceptions of transport mobility are needed. Transport policymakers have begun to associate the ability to be mobile with having a role in the facilitation of social inclusion [21]. Cities increasingly look to cycling to promote urban sustainability, liveability, and public health [22]. Light Rapid Transport is providing a significantly better quality of life compared to buses [23]. The public transport service significantly affects quality of life [24,25]. The opening of new subway lines will lead to a decline in the number of vehicles and other traffic on the roads. There is a significant rise in subway commuting trips, while nonmotorized and bus commuting trips are being reduced because of the new subway expansion [26]. The construction of rail transit can attract those who would usually drive, thus reducing the mitigating impact and environmental impact caused by driving and traffic congestion. Rail transit investment has increased greatly because of its potential to attract choice riders to switch from driving to transit. Metro transit development and the design of station-area neighborhoods have the potential to reduce driving and mitigate its impact on the environment, and slow the growth of traffic congestion [27]. Unfortunately, many cities do not have the space or resources to provide robust public transport systems, such as subways [28]. 

The operational efficiency of the public transportation system is the key to shortening commuting time. The rise of big data provides a means to improve the efficiency of the public transport system and build a smart city. One significant aspect of the smart cities concept is the production of sophisticated data analytics for understanding, monitoring, regulating, and planning the city [29]. The emergence of modern technology, such as shared cars, provides less traffic congestion and an environmentally friendly way of moving around [30]. Technical means can improve matching efficiency [31]. Improving transportation efficiency can not only decrease energy consumption and reduce carbon emissions, but can also accelerate people’s interactions, which will become more and more important for sustainable urban living. Basagana et al. [32] found that public transport strikes can lead to an increase in the number of private vehicle trips, which in turn can increase air pollution levels, and alterations in public transport have consequences for air quality. Stiglic et al. [31] found that the integration of a ride-sharing system and a public transit system can significantly enhance mobility and increase the use of public transport. Beijing-based research found no significant associations between average commuting time and the variables of local public transport accessibility and private vehicle transport accessibility. Improving the job–housing balance through the implementation of compact land development may be an alternative to reducing overall commuting duration [33]. The higher the job–housing balance, the shorter the worker’s commuting time [34]. More balanced land use improves the probability of commuting by motorcycle and electric bike but reduces the probability of commuting by public transit [35]. Urban planning and policy that promote mixed land use and job–housing balance should be considered. 

## 3. Data and Empirical Results

Next, we will empirically examine the relationship between commuting and quality of life. The data used in this study are from the 2016 China Labor-force Dynamic Survey (CLDS). The CLDS was launched by Sun Yat-Sen University in China. The survey is conducted every two years. These data provide a detailed survey of the workforce. The CLDS focuses on the working-age population aged 15-64 and focuses on the current situation and changes in the labor force, such as education, employment, labor rights, occupational mobility, occupational protection and health, and occupational satisfaction and well-being. At the same time, it is a large-scale interdisciplinary follow-up survey on the political, economic, and social development of the community where the labor force is located, the demographic structure, household property and income, household consumption, household donation, rural household production and land, etc. The 2016 CLDS sample covered 29 provinces and cities in China, with a sample size of 401 villages, 14,226 households, and 21,086 individuals.

Among them, the questions about commuting time are measured through the following question:

*In your current or recent work, the time spent on the road to work and off work every day is a total of how many minutes?* We used this question to investigate the commuting time of the workforce. To measure commuting time more accurately, we processed the sample. First, we only kept the 16–64-year-old category of working people at the city level. In addition, we removed some commute time outliers.

We used the Probit model to investigate:

*y_ic_* = *α* + *βcommuting time_ic_* + *γZ_ic_* + *ε_ic_*

The explanatory variables, *y_ic_*, are the subjective feelings related to the commuting of individual *i* in city *c*. We used life satisfaction and job satisfaction to measure the subjective feelings. In addition, we also checked health damage caused by commuting. The core explanatory variable is commuting time. *Z_ic_* are the individual-level and city-level control variables. At the individual level, we controlled age, gender, education, marital status, and income level; at the city level, we controlled per capita GDP, house prices, and population size; in terms of transportation factors, we controlled road area, number of private cars, availability of taxis, bus coverage, and subway length.

### 3.1. Descriptive Statistics

**Figure 1:**
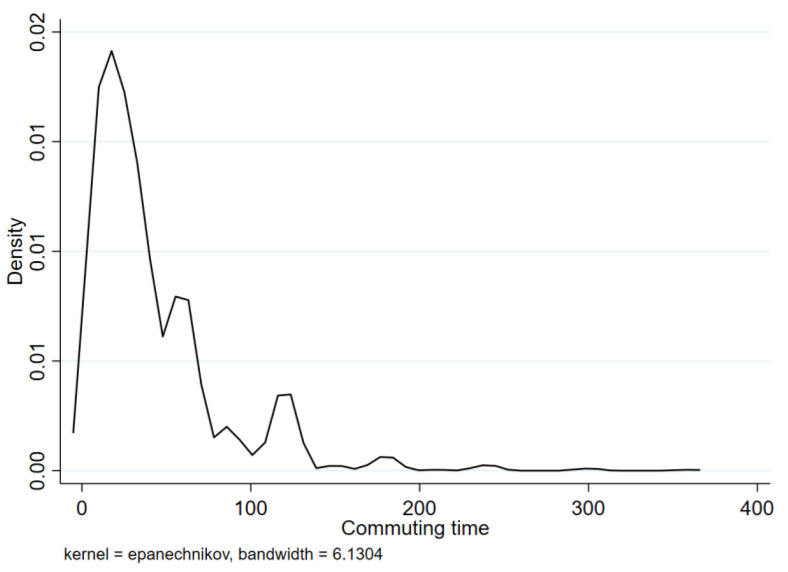
Distribution of commuting time.

**Figure 2:**
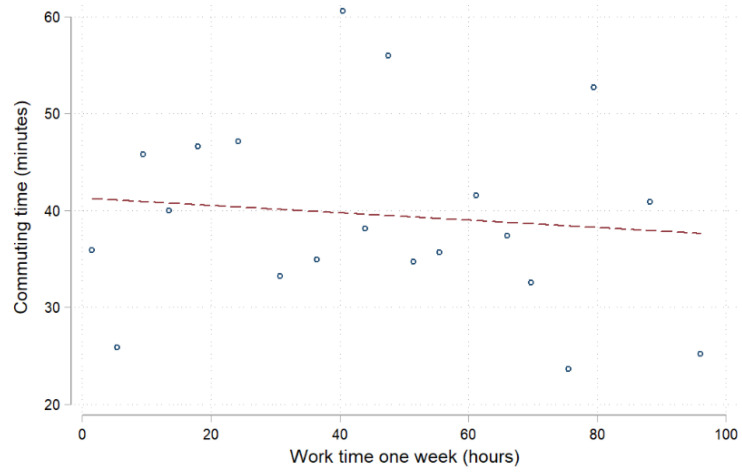
Commuting time and work time for one week.

Figure 1 shows the distribution of commuting time to and from work. From the distribution point of view, there is a difference in commuting time between individuals, but the focus is within 100 min, which is within 50 min each way. Comparing the commute time with the number of working hours per week, it can be seen that the relationship between working hours and commuting time is weak, and the variation is relatively large (see Figure 2). This shows that the difference in commuting time between different types of work is not particularly large and is mainly reflected in individual differences.

The average commute time in the data is 41.8 min, and the average one-way time is more than 20 min, which is similar to the commuter research data available in developed countries. However, the fluctuations are relatively large, and the maximum one-way commute time is 3 h.

### 3.2. Transportation Methods and Commuting

First, we discuss the relationship between commuting time and quality of life. In terms of metrics, we divided the quality of life into two categories. One is satisfaction with life, and the other is economics, that is, income satisfaction. The values are from 1–5, and the higher the value, the higher the satisfaction. This subjective satisfaction can measure the impact of commuting time on it. Table 1 reports the results of the regression. Columns (1) and (2) of Table 1 report the impact on life satisfaction. From the regression results, it can be seen that the increase in commuting time significantly reduces life satisfaction. The regression results in columns (3) and (4) are more direct indications of their impact on job satisfaction. Those with longer commutes have lower job satisfaction. Work and life are important components of quality of life. The regression in Table 1 indicates that the longer the commuting time, the worse the quality of life. From the perspective of control variables, income and education levels can significantly improve the quality of life; marriage helps improve the quality of life but does not help to improve job satisfaction; the impact of age is not significant. Considering the control variables at the city level, the more economically developed the region, the higher the satisfaction, but the higher the housing price, the higher the cost of living, and the lower the life and job satisfaction. The greater the population size, the higher the satisfaction, which shows the benefits of big cities. Table 2 shows that the influence coefficient of commuting has increased after controlling the factors at the city level.

What is the reason for the decline in life satisfaction caused by commuting time? We believe that an important factor in both the cause and the result is health. The increase in commuting time means a lot of time spent on the road, leading to the loss of mental and physical health for the workforce. We considered three variables: one is the overall perception of the personal health level; the other two are pain and concentration. Table 3 reports these results. The larger the value of these reports, the worse the health. As can be seen from the regression of Table 3, the longer the commuting time and the worse the health level, the more likely it is that the body will feel tired, and concentration will decrease. This shows that those who spend more time commuting have a health loss. Although income levels can alleviate the deterioration of health, they do not relieve pain and attention. Men receive more damage. Education level has no significant impact. The regression results at the city level show that the more developed the economy, the better the health level. Housing prices will worsen the health condition. Part of the reason here is that high housing prices worsen the job–housing balance, and increase the amount of commuting, which in turn leads to the negative effects. The city size also has a positive effect on quality of life; the increase of population makes people healthier and less tired. This may be because the bigger cities have more medical resources.

The regression results in Table 1 and Table 3 illustrate the negative impact of commuting on quality of life. Those who have been commuting for a longer period face lower life satisfaction and health. Based on this, it is important to improve the quality of life and reduce the negative factors brought by commuting time. Although research shows that the average commute time between cities does not change much, the differences across individuals are large. 

### 3.3. Commuting Time and Happiness in Life

The question of how the public transport system can improve individual commuting time thus arises. Here, we consider four forms of public transport: bus coverage, subway mileage, private cars, and taxis. In Table 4, we found that subway mileage and bus coverage significantly reduce commuting time, while taxis increase time. A possible explanation here is that those who live in remote areas and do not have bus coverage need to take a taxi. From the perspective of individual characteristics, the older the commuter and the higher the education level, the longer the commuting time.

It can be seen from the above analysis that the improvement of the public transportation system can effectively reduce the commuting time, thus improving the quality of life. Therefore, unlike the research in the existing literature, we conclude that the construction of public transportation has a significant impact on commuting time. In other words, given the current living conditions, the construction of the bus can shorten the commute time, and the speed increase may also shorten the commute time.

In Table 1 we discuss the relationship between commute time and resident satisfaction. In fact, the length of the commute depends not only on the individual’s choice of place of residence and work, but also on traffic conditions. If roads are congested, residents will have a longer commute. In Table 5, we examine the relationship between the quality of life of residents and traffic congestion. First, in Table 5, column (1), we find that traffic congestion significantly increases commuting time. On average, the traffic congestion index increased by 0.1 (average 1.65), and residents commute to work by 2.7 min (average 41.8 min). In Table 5, columns (2)–(5), we examine the impact of traffic congestion on life satisfaction and economic satisfaction. We find that the higher the congestion index, the lower the life satisfaction and economic satisfaction of the residents. Table 5 shows that congestion increases residents’ commute time and further reduces residents’ quality of life.

## 4. Conclusions and Discussion

Using the micro-survey data of CLDS, this study examines the impact of commuting time on life satisfaction and health in China. Second, we examine the effect of public transportation on commuting time. This study finds that the extension of commuting time is not conducive to the improvement of residents’ quality of life. However, improvements in public transportation can effectively decrease commuting time. 

Smart cities are increasingly focusing on becoming greener, healthier, and more sustainable. Public transport is an important path to improve mobility and reduce commuting time. The role of public transport in improving the quality of life is twofold. First, given the urban structure, efficient and fast public transport can reduce commuting time and improve travel efficiency. Second, the coverage of public transport can also ease the separation of job and housing. 

The application of big data is an important step to improve the quality of life in the future. Using big data can help accurately identify urban structures and ease the separation of job and housing. These form the basis of housing policy and resource allocation in public transport. Some new developments, such as car and bike sharing, have improved the efficiency of urban operations and people’s living standards. In policy, the application of these technologies in urban transport should be encouraged.

## Figures and Tables

**Table 1:** Commuting and quality of life.

	(1)	(2)	(3)	(4)
	Life Happy	Life Happy	Economic Happy	Economic Happy
Commuting	−0.00123 **	−0.00128 **	−0.00148 ***	−0.00165 ***
	(0.000577)	(0.000614)	(0.000566)	(0.000602)
Age	0.00122	0.000329	0.000316	−0.000369
	(0.00245)	(0.00262)	(0.00240)	(0.00257)
Marriage	0.277 ***	0.241 ***	0.0596	0.0395
	(0.0587)	(0.0631)	(0.0577)	(0.0620)
Man	−0.135 ***	−0.137 ***	−0.0968 **	−0.0999 **
	(0.0446)	(0.0480)	(0.0437)	(0.0470)
Ln (income)	0.112 ***	0.139 ***	0.191 ***	0.219 ***
	(0.0281)	(0.0320)	(0.0278)	(0.0316)
Education	0.0303 ***	0.0262 **	0.0494 ***	0.0498 ***
	(0.00976)	(0.0105)	(0.00959)	(0.0104)
Ln (per GDP)		0.0342		0.145 **
		(0.0704)		(0.0692)
Ln (house price)		−0.214 ***		−0.276 ***
		(0.0712)		(0.0700)
Ln (population)		0.142 ***		0.0617 *
		(0.0378)		(0.0370)
Industry dummy Work location	Yes Yes	Yes Yes	Yes Yes	Yes Yes
Observations	2735	2395	2735	2395

Note: *** *p* < 0.01, ** *p* < 0.05, * *p* < 0.1.

**Table 2:** Descriptive statistics.

Variable	Obs	Mean	Std. Dev.	Min	Max
*Personal level*					
Commuting	2817	41.867	40.310	1	360
Age	2817	39.924	10.360	17	64
Marriage	2817	0.800	0.400	0	1
Man	2817	0.539	0.498	0	1
Ln (income)	2817	10.466	0.825	1.609	14.509
Education	2816	5.654	2.680	1	11
Life happy	2815	3.785	0.878	1	5
Economic happy	2815	3.314	1.005	1	5
Health	2812	2.09h4	0.805	1	5
Tired	2812	1.713	0.955	1	5
Focus	2812	1.516	0.746	1	4
*City level*					
Ln (road)	2817	3.654	1.020	1.176	5.658
Ln (car)	2476	6.855	0.985	4.801	8.390
Ln (taxi)	2817	2.044	1.019	0.315	4.238
Bus coverage	2817	0.732	0.128	0.33	1
Subway length	2817	2.428	2.475	0	6.451
Ln (per GDP)	2817	11.155	0.542	9.840	11.999
Ln (house price)	2470	8.953	0.587	7.898	10.432
Ln (population)	2817	15.612	0.712	13.997	17.177

**Table 3:** Commuting time and quality of life.

	(1)	(2)	(3)	(4)	(5)	(6)
	Health	Health	Tired	Tired	Focus	Focus
Commuting	0.00179 ***	0.00153 **	0.00157 **	0.00192 ***	0.00117 *	0.00131 **
	(0.000584)	(0.000621)	(0.000611)	(0.000648)	(0.000624)	(0.000664)
Age	0.0228 ***	0.0244 ***	0.0138 ***	0.0149 ***	−0.00126	−0.000822
	(0.00250)	(0.00268)	(0.00267)	(0.00284)	(0.00273)	(0.00291)
Marriage	0.0121	−0.00119	−0.0652	−0.101	−0.0506	−0.0586
	(0.0600)	(0.0644)	(0.0642)	(0.0687)	(0.0653)	(0.0701)
Man	−0.0707	−0.0623	−0.122 **	−0.155 ***	−0.0697	−0.0761
	(0.0451)	(0.0485)	(0.0482)	(0.0518)	(0.0491)	(0.0528)
Ln (income)	−0.0936 ***	−0.101 ***	−0.0370	−0.0147	0.0271	0.0361
	(0.0286)	(0.0325)	(0.0304)	(0.0348)	(0.0311)	(0.0356)
Education	0.00639	0.00380	0.00895	0.00914	−0.0137	−0.0148
	(0.00989)	(0.0107)	(0.0106)	(0.0114)	(0.0108)	(0.0117)
Ln (per GDP)		−0.0786		−0.216 ***		−0.218 ***
		(0.0712)		(0.0758)		(0.0773)
Ln (house price)		0.320 ***		0.287 ***		0.290 ***
		(0.0722)		(0.0773)		(0.0781)
Ln (population)		−0.184 ***		−0.141 ***		−0.117 ***
		(0.0383)		(0.0409)		(0.0417)
Industry dummy	Yes	Yes	Yes	Yes	Yes	Yes
Work location	Yes	Yes	Yes	Yes	Yes	Yes
Observations	2732	2392	2732	2392	2732	2392

Notes: *** *p* < 0.01, ** *p* < 0.05, * *p* < 0.1.

**Table 4:** Transport and commuting time.

	(1)	(2)	(3)
	Commuting	Commuting	Commuting
Age	0.00795 ***	0.00778 ***	0.00666 **
	(0.00230)	(0.00246)	(0.00261)
Marriage	−0.128 **	−0.0706	−0.0590
	(0.0553)	(0.0595)	(0.0628)
Man	−0.129 ***	−0.0925 **	−0.114 **
	(0.0419)	(0.0451)	(0.0475)
Ln (income)	0.0217	−0.0589 *	−0.0422
	(0.0265)	(0.0300)	(0.0316)
Education	0.0567 ***	0.0570 ***	0.0563 ***
	(0.00918)	(0.00989)	(0.0106)
Ln (per GDP)		0.169 **	0.391 ***
		(0.0663)	(0.106)
Ln (house price)		0.00756	−0.139 *
		(0.0670)	(0.0795)
Ln (population)		0.106 ***	0.140
		(0.0356)	(0.0930)
Ln (road)			−0.0483
			(0.0532)
Ln (car)			−0.119
			(0.0962)
Ln (taxi)			0.295 ***
			(0.0582)
Bus coverage			−0.566 *
			(0.326)
Subway length			−0.0377 *
			(0.0220)
Industry dummy	Yes	Yes	Yes
Work location	Yes	Yes	Yes
Observations	2737	2397	2168

Notes: *** *p* < 0.01, ** *p* < 0.05, * *p* < 0.1.

**Table 5:** Congestion and quality of life.

	(1)	(2)	(3)	(4)	(5)
	Commuting	Life Happy	Life Happy	Economic Happy	Economic Happy
Congestion index	27.13 ***	−0.378	−0.497 *	−0.937 ***	−1.011 ***
	(8.156)	(0.234)	(0.287)	(0.230)	(0.281)
Age	0.350 ***	0.00498 *	0.00213	0.00250	0.000582
	(0.0968)	(0.00277)	(0.00295)	(0.00272)	(0.00290)
Marriage	−6.573 ***	0.254 ***	0.240 ***	0.0482	0.0301
	(2.291)	(0.0653)	(0.0701)	(0.0643)	(0.0690)
Man	−2.548	−0.121 **	−0.129 **	−0.0711	−0.0991 *
	(1.743)	(0.0499)	(0.0531)	(0.0489)	(0.0521)
Ln (income)	0.598	0.0756 **	0.109 ***	0.179 ***	0.213 ***
	(1.130)	(0.0323)	(0.0355)	(0.0319)	(0.0351)
Education	1.702 ***	0.0346 ***	0.0285 **	0.0554 ***	0.0512 ***
	(0.384)	(0.0110)	(0.0117)	(0.0108)	(0.0115)
Ln (per GDP)			−0.171 *		0.0229
			(0.0942)		(0.0924)
Ln (house price)			−0.111		−0.237 ***
			(0.0818)		(0.0805)
Ln (population)			0.179 ***		0.178 ***
			(0.0476)		(0.0469)
Constant	−39.19 **				
	(17.99)				
Industry dummy	Yes	Yes	Yes	Yes	Yes
Work location	Yes	Yes	Yes	Yes	Yes
Observations	2152	2150	1916	2150	1916
R-squared	0.238				

Notes: *** *p* < 0.01, ** *p* < 0.05, * *p* < 0.1. The congestion index is from Gaode, a map service company, which publishes a daily traffic congestion index for 100 cities.
